# Supplementary figures and images for: Impact of hysterectomy on opioid use in patients with adenomyosis: A nationwide register study
Source: PLoS One. 2025 Jan 15;20(1):e0317135. doi: 10.1371/journal.pone.0317135 (PMC11734910; doi:10.1371/journal.pone.0317135)

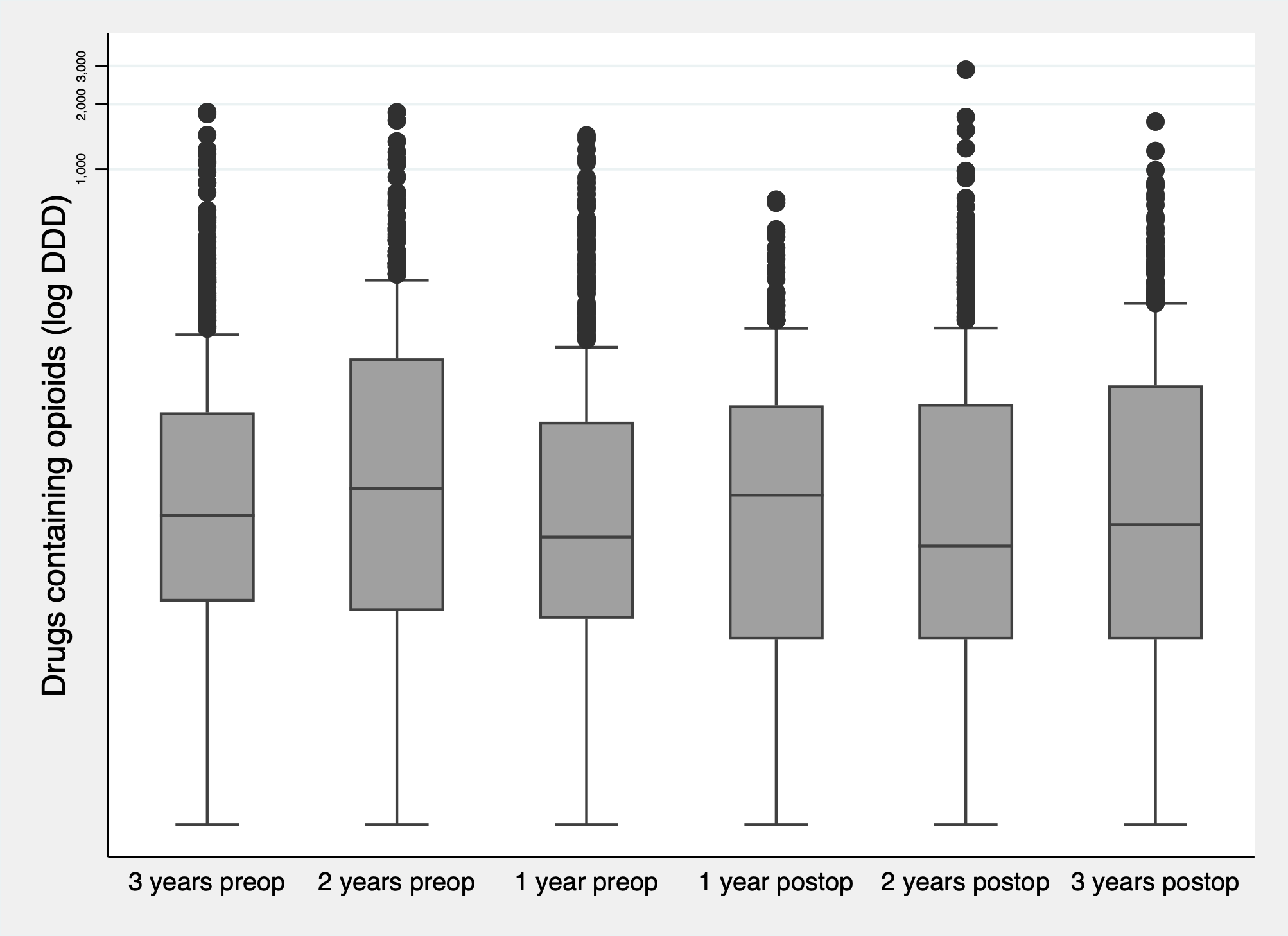

Supplement: S1 Fig — y-axis: frequency of World Health Organization Defined Daily Dose. x-axis: preop = preoperatively, postop = postoperatively. Patients with adenomyosis. 3 years before compared to 3 years after hysterectomy (IRR 1.1 95% CI 1.0–1.3). (TIF) [file pone.0317135.s001.tif]

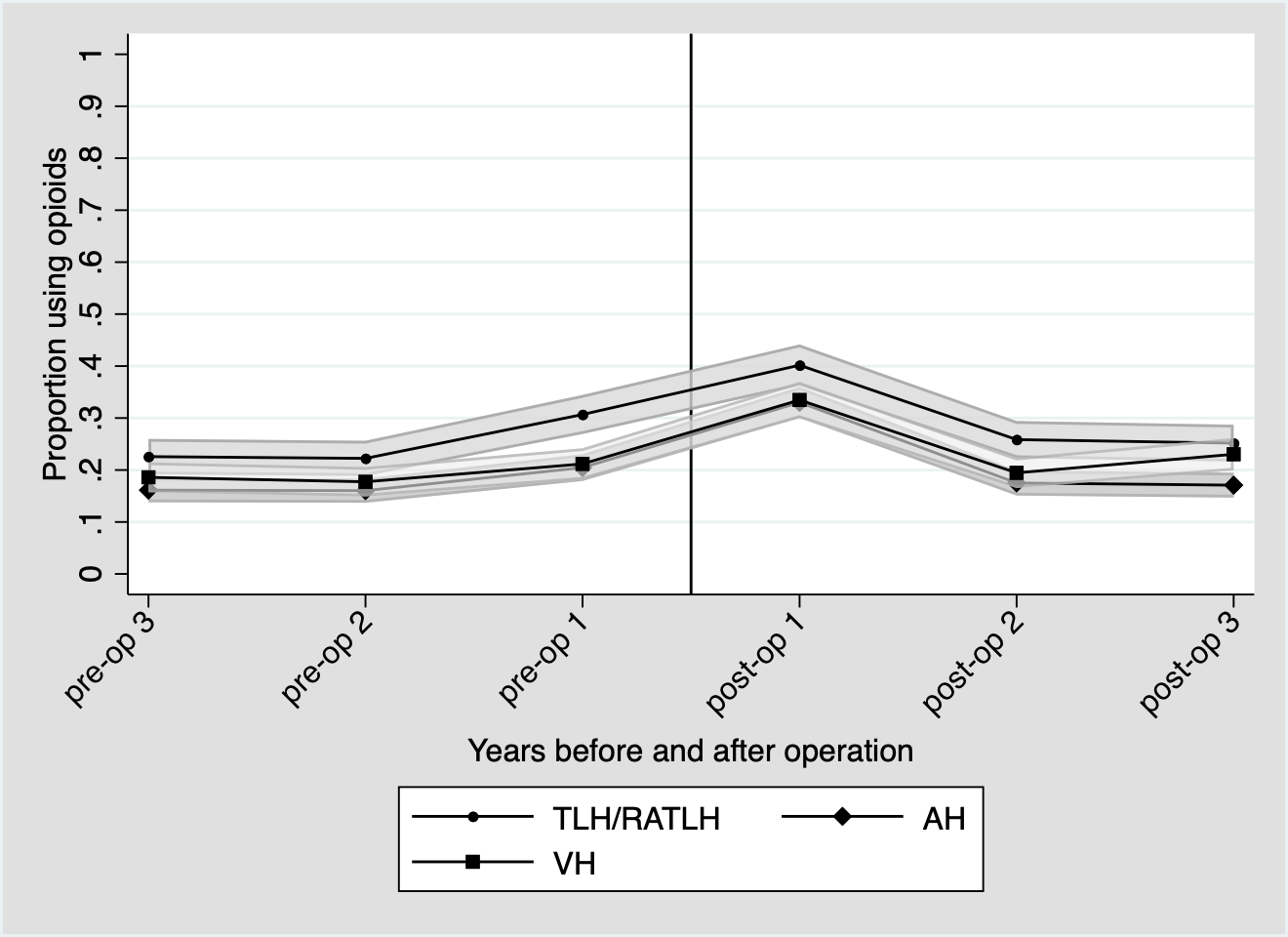

Supplement: S2 Fig — Analyze of interaction in proportion of opioid users including hysterectomy procedure and study year (base outcome vaginal hysterectomy and 3 years preoperative). Graph showing point estimate and 95% confidence interval (shadowed area). TLH/RATLH = Laparoscopic/Robotic assisted hysterectomy AH = Abdominal hysterectomy VH = Vaginal hysterectomy. Preop = Before hysterectomy; Postop = After hysterectomy. OR, Odds Ratio; 95% CI, 95% Confidence Interval. a = adjusted for baseline data including all variables from Table 1. (TIF) [file pone.0317135.s002.tif]

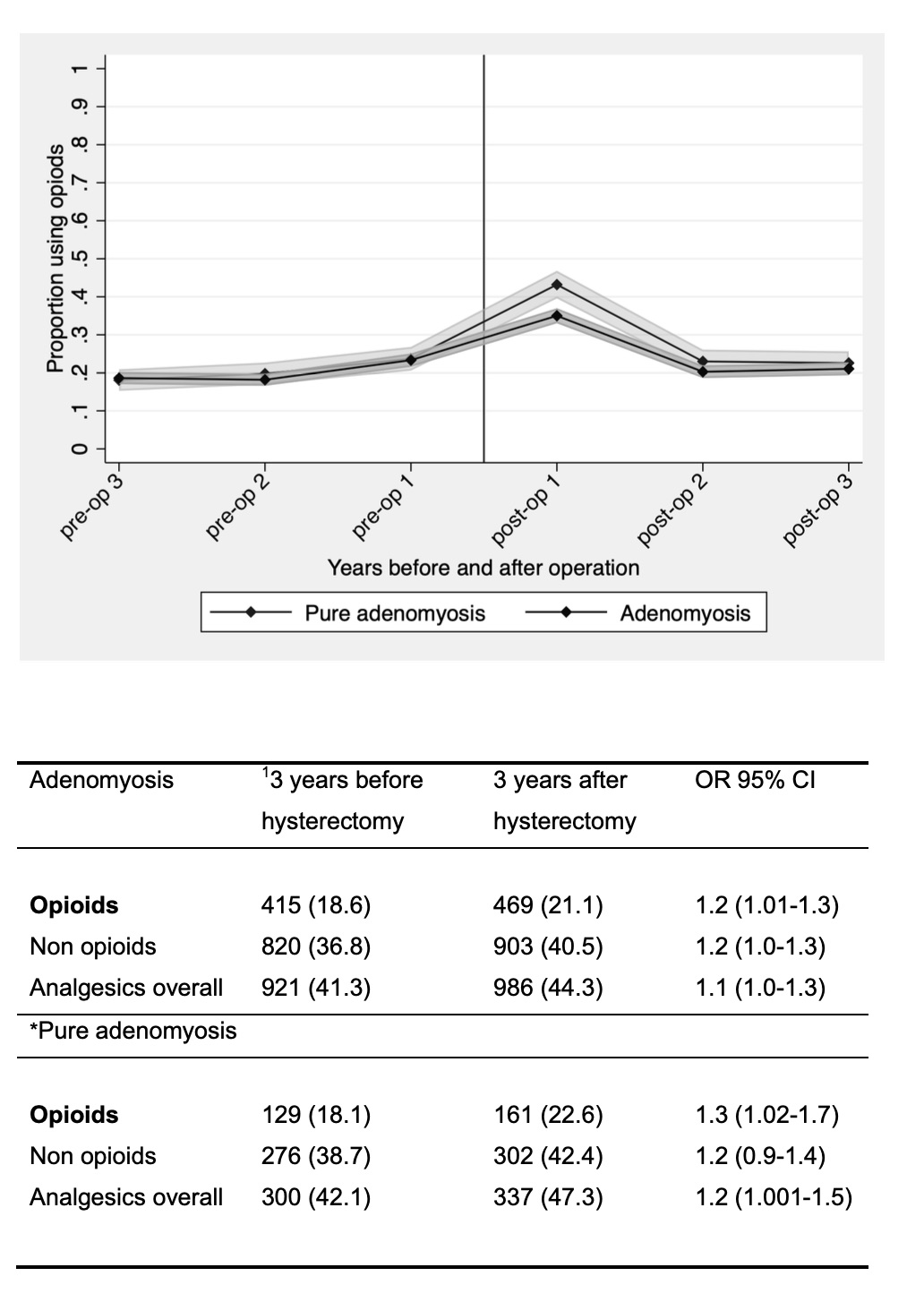

Supplement: S3 Fig — Graph showing point estimate and 95% confidence interval (shadowed area). Preop = Before hysterectomy; Postop = After hysterectomy. 1Reference. Data are presented as frequencies (proportions). OR = odds ratio, CI = confidence interval. Non opioids = Paracetamol, Aspirin, non steroid anti-inflammatory drugs. *Pure adenomyosis = only patients with post-operative pathology main diagnoses of adenomyosis (co-diagnoses adenomyosis excluded) + any diagnosis of endometriosis was excluded. (TIF) [file pone.0317135.s003.tif]
