# Supplementary material for: Impact of hysterectomy on opioid use in patients with adenomyosis: A nationwide register study
Source: PLoS One. 2025 Jan 15;20(1):e0317135. doi: 10.1371/journal.pone.0317135 (PMC11734910; doi:10.1371/journal.pone.0317135)
Supplement: S1 Table — (DOCX) [file pone.0317135.s004.docx]

**S1 Table. A description of how drugs were grouped from the Swedish Prescribed Drug Register.**

| Drug group | ATC code |
| --- | --- |
| ^4^Hormonal treatment for endometriosis (including contraceptives) | G03A, G03X, G02B, L02A, H01C, L02B and G03D |
| Hormonal treatment for menopausal symptoms | G03C and G03F |
| ^1^Opioids | N02A |
| ^1^Non-opioids analgesics | M01A, M02A, N02B and M01B |
| ^1^Muscle relaxants | M03B |
| ^2^Anti-depressants | N06A |
| ^2^Sedatives and sleeping pills | N05B and N05C |
| ^3^Neuroleptic drugs | N05A |
| ^3^Antiepileptic drugs | N03A |
| ^3^Psycho-stimulants | N06B |

^1^Analgesics
^2^Psychoactive drugs
^3^Neuroactive drugs

ATC code = Anatomical Therapeutic Chemical Classification System

^4^Levonorgestrel intrauterine device (IUD) was included in this group, however there were no data on when the IUD was removed
