# Supplementary material for: Impact of hysterectomy on opioid use in patients with adenomyosis: A nationwide register study
Source: PLoS One. 2025 Jan 15;20(1):e0317135. doi: 10.1371/journal.pone.0317135 (PMC11734910; doi:10.1371/journal.pone.0317135)
Supplement: S2 Table — (DOCX) [file pone.0317135.s005.docx]

**S2 Table. S2 Fig data.**

|  | VH | Interaction  AH and study year  cOR (95%CI) | Interaction  AH and study year  aOR (95%CI) | Interaction TLH/RATLH  and study year  cOR (95%CI) | Interaction TLH/RATLH  and study year  aOR (95%CI) |
| --- | --- | --- | --- | --- | --- |
| Opioids | Reference | 0.8 (0.6-1.2) | 0.8 (0.4-1.5) | 0.9 (0.6-1.3) | 0.8 (0.4-1.6) |

Graph showing point estimate and 95 % confidence interval (shadowed area).

TLH/RATLH = Laparoscopic/Robotic assisted hysterectomy AH = Abdominal hysterectomy VH = Vaginal hysterectomy

Preop = Before hysterectomy; Postop = After hysterectomy

OR, Odds Ratio; 95% CI, 95% Confidence Interval

a = adjusted for baseline data including all variables from Table 1
