# Supplementary material for: Impact of hysterectomy on opioid use in patients with adenomyosis: A nationwide register study
Source: PLoS One. 2025 Jan 15;20(1):e0317135. doi: 10.1371/journal.pone.0317135 (PMC11734910; doi:10.1371/journal.pone.0317135)
Supplement: Attachment — Submitted filename: Response to Reviewers.docx [file pone.0317135.s006.docx]

Dear Editor,

We are pleased that the reviewers helped us to improve this manuscript through their valuable suggestions, and that you consider our article for publication in your journal. We have read and appreciated the comments from the reviewers, please see our responses below.

Response to Reviewers

Dear reviewers,

We are very grateful for your valuable comments that really have helped us improve our paper.

Reviewer #1: Dear authors, please find my comments to your manuscript here:

**1.- please comment on the fact that only 3.9 % of patients with adenomyosis have had an indication for hysterectomy because of adenomyosis. Did you analyze the indication in the other 96.1 % of patients with adenomyosis?**

Thank you for a very relevant remark. In Sweden, the indication for the surgical procedure is identified as the diagnosis the patient received on the day of surgery. What we mean here, is that only 3,9% of patients who had a hysterectomy had a **main** diagnosis of adenomyosis on the day of the hysterectomy (other adenomyosis patients might have had “menorraghia” as main diagnosis, for example).

Firstly, we believe that the low rate of correct pre-op diagnosis is worth highlighting due to the (hopefully, former) low awareness of adenomyosis and the corresponding signs on TVUS.

Secondarily, many patients are scheduled for hysterectomy in Sweden with symptom-related diagnosis only, where adenomyosis might be the underlying cause, for example ”menorraghia”(36%) or ”dysmenorrhea”(1.5%). Also the large group of ”myoma” (18.9%) might include misdiagnosed adenomyosis/adenomyoma, and 30-40% of the adenomyosis patients did indeed also have myomas and/or endometriosis (2,7% of total indications). 29,5% of indications were unfortunately missing.

We have now added this information in Results section for clarification, as below (line 216-219:

Before surgery, 86 of 2,228 patients (3.9%) had adenomyosis registered as main indication for surgery. Main registered indications for hysterectomy (on the day of surgery) in this dataset includes “menorraghia”(36%), “myoma” (18.9%), “dysplasia”(2.8%), “adnexal pathology”(2.7%), “endometriosis”(2.7%) and “dysmenorrhea”(1.5%), and the rate of missing data was 29.5%.

**2.- In Table 1 you added info on additional Endometriosis. But there is no differentiation of peritoneal and deep endometriosis and the type and localization. Do you think that a reason for continuous opioid use could be underdiagnosed and therefore undertreated DE? Missed bowel endometriosis? etc.**

Thank you for this valid point. The reason of the paucity of endometriosis differentiation, is that the #Enzian score (which is now mandatory part of the register) was not established at the time of the study. In the dataset presented here, the amount of missing data on endometriosis differentiation was 43% and 47% in the two groups of adenomyosis patients, a level we consider too high to use in an analysis. There is however a statistically low risk of significantly underdiagnosed deep endometriosis in the long-term group compared to the no long-term group, since the proportion of co-current endometriosis is almost exactly the same in the two groups (15,4% and 15,9%, respectively). This has now been clarified in the Result section, as below (line 316-318):

Neither parity, BMI, smoking or previous abdominal surgery was significantly associated with long-term postoperative consumption of opioids, neither was the prevalence of endometriosis (15.4% in long-term users vs 15.9%) or the use of preoperative hormonal treatment (69.6% in long-term users vs 63.8%) (table 1).

And in Discussion section (line 367-379):

We acknowledge the lack of systematic endometriosis differentiation at the time of the study (i.e the #Enzian score, which is since June 2023 a mandatory part of The Swedish National Quality Register of Gynecological Surgery). Although this is a weakness of this investigation, we consider the risk of undiagnosed deep endometriosis similar in patients with or without long-term opiod treatment, since the frequency of co-current endometriosis was almost exactly the same in both groups.

**-3. Please comment on the possible lack of detection of additional deep endometriosis in the time of the study (2012-2015) (there current role of pre surgical imaging was less common at that time).**

It is definitely also our experience, that pre-surgical imaging was less systematic regarding endometriosis in the years of the study. To aid us in future studies, we have now added the #Enzian score of endometriosis as mandatory in the national register of gynecological surgery. We are hoping for better pre-surgical imaging also of adenomyosis (as it is also included in the #Enzian score) in the future. Please see Discussion section for additional comments (stated in question 2 above).

**-4. Line 302: please explain your speculation. In my opinion there is no clear explanation for your assumption. If you have no reference, this part should be omitted.**

We understand your remark, and have omitted this speculation as suggested.

~~One could speculate that adenomyosis with a lesser extent of co-current myomas (30,6% in long-term opioid users, vs 40,2% in no long-term users, table 1) is a more inflammatory and painful condition than a combined disease, which could possibly explain more pain pre-operatively.~~

**-5. Line 311: as mentioned above, this should be discussed in detail.**

Thank you for this remark. The reference of endometriosis prevalence is from the register, however we have now extended the discussion, mentioning the lack of detailed endometriosis diagnostics at the time of study. Please see response to questions 2 for corresponding changes in the manuscript.

**6.- Line 315: please explain if a guideline for histopathological workup of hysterectomy specimens in search for adenomyosis exists in Sweden? Would you think it's possible, that the proportion of adenomyosis could be much higher, because pathologists missed the disease? Please discuss.**

We totally agree that the low level of detected adenomyosis is due to low awareness – but it might also be due to the operating gynecologist, and not only the pathologist – if the surgeon has a low awareness and do not ask for presence of adenomyosis, the chance of a thorough histopathology examination in this regard is probably lower. In Sweden all uteri with suspected benign disease are examined macroscopically by the pathologist, followed by 2 biopsies from cervix uteri and 4 biopsies from corpus uteri, and additional biopsies from myomas >5cm. From macroscopically suspected areas (such as overt adenomyosis) additional biopsies are taken. Only in suspected malign disease, 5mm slices are used. We have added a comment on the pathology routine in the Material&Methods as well as in the Discussion section, please see below:

M&M line 132-135: In Sweden all uteri with suspected benign disease are examined macroscopically by the pathologist, followed by 2 biopsies from cervix uteri and 4 biopsies from corpus uteri, and additional biopsies from myomas >5cm. From macroscopically suspected areas (such as overt adenomyosis) additional biopsies are taken.

Discussion line 387-391: The Norwegian Adenomyosis Study also included a more thorough pathology protocol (including 5mm-slices of uterus, which is comparable to the Swedish protocol for suspected malign uterine disease) where the gynecologist responsible for the pre-operative diagnose also participated, which maximized the sensitivity (15), but does not represent the clinical setting in most countries, including ours.

**7.- Your conclusion gives the impression that hysterectomy does not decrease dysmenorhoe and pain in patients with adenomyosis. In my opinion, your results can not support this assumption and therefore this fact should be made very clear in the conclusion and the abstract.**

Thank you for this remark. We have now clarified that opioid use is a proxy for pain and other conditions affecting opioid use is unknown. We have furthermore clarified the lack of data on self-reported pain post-operatively, which is of course a weakness of the study. We have now made clarifications in the abstract, the Discussion and in the Conclusion section, as shown below:

Abstract line 67: Hysterectomy does not reduce use of opioids among adenomyosis patients in long-term follow up, although other conditions affecting opioid use were not known.

Discussion line 347-348: This study shows that hysterectomy was not associated with a post-operative reduced opioid use in a population of patients with adenomyosis.

line 405-407

Furthermore, the missing data from patient-reported questionnaires was too high to include analysis of subjective pain levels pre- and post-operatively, which resulted in the analysis of opioid use as a proxy for pain. Therefore, we cannot conclude that the opioid use is due to pain caused by adenomyosis, but simply acknowledge that the use does not decline after hysterectomy.

Conclusion line 418: Hysterectomy does not reduce use of opioids post-surgery in patients with adenomyosis, although other condition affecting opioid use was not known.

**8. In my opinion, the most important limitation of your work is the fact, that you look at adenomyosis without looking at endometriosis. Of course, adenomyosis can be found without additional peritoneal or deep endometriosis, but in many patients (references) both conditions can be found simultaneously. This should be discussed.**

We absolutely agree with the reviewer that the differentiation of endometriosis is clearly suboptimal in this dataset, as we added to the discussion as suggested above (see question 2 and 3). The #Enzian score will hopefully aid better research in the future. To clarify, we have added to the Result section the similar levels of co-existing endometriosis diagnoses in the two adenomyosis groups (15.4% and 15.9%, respectively) as stated in question no 2.

To further address the possible characteristics of the group of adenomyosis **only** (here described as “pure adenomyosis”, vs adenomyosis+concomitant condition such as endometriosis and/or fibroids) we have added a supplementary figure, where it is shown that there was no significant statistical difference between these two groups, regarding levels of opioid use pre- and post surgery:

This figure is added in the Result section together with the following text, to address this remark (line 324-327) :

To address possible characteristics of adenomyosis only (no concomitant endometriosis and/or myomas) vs adenomyosis as additional diagnosis, we performed a subgroup analysis (fig S3) showing no significant difference in opioid use between these two groups.

**Reviewer #2:**

**Thank you for this clear manuscript of an important issue.**

Thank you very much for that comment!
 **ABSTRACT
1. Methods: retrospective/historical register-based study?**

We agree with the reviewer that this is a retrospective register-based cohort study, and this is now clarified in the abstract (line 52): This is a nationwide retrospective register-based cohort study

**2. Results: lines 51-54: .. preoperative use of opioids and antidepressants
Conclusions: Should be more in line with the results. Hysterectomy did not reduce … in our study. .. However, other conditions affecting opioid use were not known.**

Thank you for this remark, this has been clarified as suggested (line 67): Hysterectomy does not reduce use of opioids among adenomyosis patients in long-term follow up, although other conditions affecting opioid use were not known.

**INTRODUCTION
3. Page 5, line 77: “>160 years later “– rather “more than 160 later..”?** This is now corrected.

**MATERIAL AND METHODS
4.- I would like to know all medications you have retrieved to your study. I suggest you add a supplementary table including all ATC codes and affecting agents you have included in this study.**

Thank you for the valuable suggestion, the table suggested is now added (as below) in the Material&Methods section:

**Supplementary table.** A description of how drugs were grouped from the Swedish Prescribed Drug Register.

| **Drug group** | **ATC code** |
| --- | --- |
| ^4^Hormonal treatment for endometriosis (including contraceptives) | G03A, G03X, G02B, L02A, H01C, L02B and G03D |
| Hormonal treatment for menopausal symptoms | G03C and G03F |
| ^1^Opioids | N02A |
| ^1^Non-opioids analgesics | M01A, M02A, N02B and M01B |
| ^1^Muscle relaxants | M03B |
| ^2^Anti-depressants | N06A |
| ^2^Sedatives and sleeping pills | N05B and N05C |
| ^3^Neuroleptic drugs | N05A |
| ^3^Antiepileptic drugs | N03A |
| ^3^Psycho-stimulants | N06B |

^1^Analgesics
^2^Psychoactive drugs
^3^Neuroactive drugs

ATC code = Anatomical Therapeutic Chemical Classification System

^4^Levonorgestrel intrauterine device (IUD) was included in this group, however there were no data on when the IUD was removed

**5.- Determine which opioids were included (mild and strong as well?)** As shown in the table, all opioids were included.

**6.- You mention muscle-relaxants here as well, were they included somewhere?** Yes, as shown in the table above. Muscle-relaxants were a (very small) proportion of the analgesics group, and were included here.

**7. - You do not mention where you have retrieved the hormonal treatment before – patient of gynecologist based data from register or from SPDR?** It comes from the SPDR, we believe this is now clarified when we added the table, again a very good suggestion.

**8.- Do you know how many of the women had levonorgestrel releasing intrauterine device preoperatively (specific ATC code)?**

Thank you for this important remark. Levonorgestrel releasing IUD was included in the hormonal treatment section, as clarified above in the newly inserted table: However, since data was retrieved from the SPDR we did not know if or when the hormonal IUD were discontinued (this is also the reason why we did not perform a subgroup analysis of this group). This is a clear weakness of this study and has been clarified under the suggested Supplementary Table 1 as well as in the Discussion section (line 403-405):

The registered data on Long-acting Reversible Contraceptives (LARC) is limited to prescription only, thus we cannot conclude when discontinuation of LARC occurred.

**9.- Did all have three year pre and post operative follow-up data surveillance with medicines? Did you have surveillance of emigrations, death – was this assessed?**

Yes, all patients have 3 years pre and post operative data on medications, they are retrieved electronically straight from all pharmacies to the register. We did not have data of emigrations and deaths, however the dataset consists of >96% individuals of ASA group 1-2, the numbers of deaths should thus be extremely low, and should be equal in both groups.

**10.- How the DDD was calculated in your study? All bought medicines in one years/365 days? Define this clearly.**

Thank you for this valuable comment. Data was collected yearly during the study period, this has been clarified in the Material&Methods section (DDD was thus calculated yearly) line 159-162:

Data on the proportion of patients using the studied medications was collected for each year of the study period. As a secondary outcome, in the opioid group, differences in the World Health Organization Defined Daily Dose (DDD) were analyzed yearly before and after hysterectomy

**11.- The subanalyses (for potential risk factor to long-term use) would have been concomitant endometriosis/solely adenomyosis since many might stop their hormonal medication after surgery**

Thanks for a very interesting remark. Surely, on can suspect adenomyosis patients to discontinue hormonal medications postop to a higher extent than patients with combined adeno/endo. Due to our curiosity regarding possible characteristics of the “pure” adenomyosis group (no concomitant endometriosis and/or myomas) when we started the study, a subgroup analysis of this group has previously been made. We did however not include this figure in the original manuscript, since there were no apparent differences between opioid use over time in pure adenomyosis patients vs adeno patients with combined disease – see the figure below. We have now once again included this figure as Supplementary (fig S3), to partly answer your (and probably others) question, together with the following text in the Results section (line 324-327):

To address possible characteristics of adenomyosis only patients (no concomitant endometriosis and/or myomas) vs adenomyosis as additional diagnosis, we performed a subgroup analysis (S3 Fig) showing no significant difference in opioid use between these two groups.

**12.- Did you assess those whose indication was adenomyosis preoperatively separately (only 86)?** No, the ethical permission did not allow us to extract individual patient records.

**13.- Exclusion criterias are presented here – prolapses excluded, why?** Prolapse consisted of only 15% of all hysterectomies during the study period, and concerns mainly post menopausal patients. Since adenomyosis rarely is symptomatic post menopaus, we chose to exclude this indication.

**RESULTS
14.- The similarity on number of endometriosis and hormonal preoper treatment between the long-users and non-users coud be mentioned here (Table 1)**

Thank you for this suggestion, it is now added as below (line 316-318):

Neither parity, BMI, smoking or previous abdominal surgery was significantly associated with long-term postoperative consumption of opioids, neither was the prevalence of endometriosis (15.4% in long-term users vs 15.9%) or the use of preoperative hormonal treatment (69.6% in long-term users vs 63.8%) (table 1).

**15. - Please mention here n(%) of the types of surgeries (AH, TLH, RTLH, VH)** Absolutely, this is now clarified in the text (line 322):

In a subgroup analysis (fig S2) we could not find any significant differences in route of hysterectomy when comparing opioid use three years before to three years after hysterectomy (in total: 948 (43%) AH, 580 (26%) TLH/RTLH, 699 (31%) VH).

**16.- How many of the used mild, how many strong opioids before and after?**

Thank you for your question. We have not separated mild and strong opioids, since the guidelines in Sweden (viss.nu) do not advocate this separation. The rationale they state, is that a high dose of mild opioids can be as strong as a low dose of strong opioids. The CDC in USA separates slow-releasing from immediate releasing opioids (cdc.gov), but they do neither separate in mild and strong opioids.

**17.- The use of other drugs in refs (in women not using opioids)?**

We are not sure that we understand this question. If you could please specify what drugs you mean, and during what interval in time, maybe we could add this in a second revision?

**18.- In which age group the long term opioids used the most?** 314 (49 %) of the patients in the long term opioid group were 45-54 years old.

**19.- Unfortunately watching
o Figure 2 The Table under OR 1.2 (95%CI 1.0-1.3 : I do not find this significantly higher, the overall use o Figure S1 and IRR 1.1 (95%CI 1.0-1.3) I do not find the latter time DDD significantly higher but not lower either.**

There are certainly no big differences in these figures (however statistically significant), therefore we do not draw any conclusions from this minimal increase - we believe the most interesting finding, is that the usage of analgesics do not decrease post hysterectomy.

**DISCUSSION
20. - Rather “This study shows that the hysterectomy was not associated with…”** Corrected accordingly

***21. - How many of the women are symptomless with adenomyosis? This might be discussed as well.***

This is a very interesting – and difficult -question. In the literature, the prevalence of adenomyosis in general population (not hysterectomy cohorts) is ranging from 5 to 70%(!), clearly representing a low concordance in diagnostic criteria’s. We do not know how many of the affected individuals that are asymptomatic in the general population. In the Swedish setting, women generally do not visit gynecologists for routine controls at all (pap-smears were previously performed by mid-wifes, and today as a self-test) - they seek gynecologists when they experience symptoms. Still, we can of course not rule out the presence of asymptomatic adenomyosis patients in this cohort, since the patient-reported questionnaires had a high level of missing data. Partly to clarify this, we have added the main indications for hysterectomy in the Results section.

**FIGURES AND TABLES
22. Difficult to understand as partly in text and partly in the end!**

We are sorry that this might be difficult to follow. However, this was the instructions for authors when creating the manuscript for PLOS ONE.

**23. Figure 1 – flowchart: dark**

Thank you for this suggestion. We agree, this issue has been corrected in Figure 1.

**24. Figure 2- Was the point estimate of persons using in preop 3 years before or the mean of preop 3 years- but up to preop 2 years (should be in methods how calculated)
If mean - your dots in the figure might be better in the middle of two time points?**

We are sorry this was not clear. The graph shows proportion of patients using opioids of each year. This has been clarified in methods (line 159-160)

**25. The table under this is not clear: Adenomyosis vs years? The references, where, should be clarified! Figure 2 is before Table 1 – the adjusted variables has to listed here**

Thank you so much for noticing this error in the manuscript. This was so helpful. We are sorry for this mistake. The text under the figure was not correct. This text has been corrected under Figure 2. When comparing prescription of drugs before and after hysterectomy the results were not adjusted for confounders since this was the same individuals and thus self-controlled. This has been described in methods line 194-197

**26. Figure 3- These figures could be joined to one.**

We tried to join these figures into one. However, according to us the graph was harder to read when joined, since the lines in the two graphs were close to each other.

**27. Figure S2- “…hysterectomy+years”? Explanations for THL/RATLH etc lacking
Table 1 Adjusted variables should be**

Thank you for this comment, we have clarified the text under figure S2 to reduce the risk for misunderstandings.
