# Supplementary material for: Impact of hysterectomy on opioid use in patients with adenomyosis: A nationwide register study
Source: PLoS One. 2025 Jan 15;20(1):e0317135. doi: 10.1371/journal.pone.0317135 (PMC11734910; doi:10.1371/journal.pone.0317135)
Supplement: Attachment — Submitted filename: Response to Reviewers Rev 2.docx [file pone.0317135.s007.docx]

**Response to Reviewers**

Dear Reviewers,

Thank you for your interest in this important subject and your valuable comments helping us improve this manuscript

Reviewer #1: Dear authors,

thank you for addressing all comments. Although you added additional information and your work is very good and very readable, I am still not fully convinced regarding your conclusion. Please let my explain my doubts:

It reads as if hysterectomy does not help patients in case of adenomyosis. There is relevant literature that shows hysterectomy to be an efficient treatment of adenomyosis related symptoms. Publications also show that patients with deep endometriosis and adenomyosis have a higher risk of re-intervention in case of previous fertility sparing deep endo excision surgery due to persistent adenomyosis symptoms.

We agree that our conclusion is not in line with some previous publications, this is discussed in line 327-332.

The fact that patients with adenomyosis might also suffer from additional under diagnosed deep endometriosis should be further discussed. Post-hysterectomy deep endometriosis is a problematic condition that could be additionally explained.

We fully agree that undiagnosed presence of deep endometriosis could be a reason of continuous pain after hysterectomy, and have now clarified the Conclusions, both in the Abstract Line 58-60:

Hysterectomy does not reduce opioid use among adenomyosis patients in long-term follow-up, although the subjective reduction of pain was not investigated in this study. Women with preoperative use of opioids/antidepressants and uterine size <300g are at increased risk for chronic opioid use.

as well as in the main manuscript, line 396-401:

Hysterectomy does not reduce long-term use of opioids post-surgery in patients with adenomyosis in this study. Subjective reduction of postoperative pain was not possible to investigate due to large amount of missing data. Risk-factors for continuous use of opioids after hysterectomy in patients with adenomyosis is pre-operative use of opioids/antidepressants and uterine size <300g.

and in discussion line 384-391:

Although the effect of hysterectomy treatment on heavy menstrual bleeding is curative, our data suggests that hysterectomy does not reduce need for opioid treatment among adenomyosis patients. However, according to what we discuss above, we cannot conclude that hysterectomy does not reduce subjective pain levels in patients with adenomyosis. Further research into which women benefit most from surgical intervention with hysterectomy for adenomyosis with a special focus on pre- and perioperative diagnosis of deep endometriosis should be a focus to improve patient counseling.

Another aspect is the opioid use itself. Why would almost 20 % of patients use opioids pre-surgically? What's the reason for that? And does the addictional aspect of opioids add to ongoing use?

We believe that the high proportion of opioid users is due to the high prevalence of chronic pain in this patient group, as well as an effect of the well-known overprescription of opioids as a treatment for chronic pain. This is discussed in line 365-370

In my opinion, it would be important to add these open questions to discuss to the conclusion and abstract. Many readers go quickly through these sections and in this case would learn: hysterectomy does not seem to work in adenomyosis related pain. And in my opinion this should not be the message of your study. Especially as the data is some years old and things changed in the meantime.

Early diagnosis, better TVS quality, less opioids, complete adenomyosis and endometriosis excision....that would make the difference.

We totally agree, and hope for: better diagnostics, carefully chosen surgical interventions and lesser opioids in the future, regarding this unfortunate patient group. This has been clarified in discussion line 384-391:

Although the effect of hysterectomy treatment on heavy menstrual bleeding is curative, our data suggests that hysterectomy does not reduce need for opioid treatment among adenomyosis patients. However, according to what we discuss above, we cannot conclude that hysterectomy does not reduce subjective pain levels in patients with adenomyosis. Further research into which women benefit most from surgical intervention with hysterectomy for adenomyosis with a special focus on pre- and perioperative diagnosis of deep endometriosis should be a focus to improve patient counseling.

Best regards.

Reviewer #2: Thank you. My questions were explained and clarified well.
I find this study important to be published.

However, jus a few minor revisions:
-I would add to Results that women aged 45-54 were the long term users who used most opioids.

We are sorry, but there was a mistake it was the age group < 45 that was the most common age group in long term users 314 (49 %) of the patients in the long term opioid group were <45 years old. However, age was not a statistically significant risk factor for long term use.

As suggested, this has been clarified in results line 271-273:

A total of 314 patients (49%) in the long-term opioid group were under 45 years old. However, age was not identified as a statistically significant risk factor for long-term opioid use.

-In addition, in Introduction I would spell out the most used opioids in Sweden (parasetamol plus codeine, tramadol?) and I would consider placing the most important opioid drugs by their general names in the Methods section.

Thank you for this valuable comment. This has been added in Introduction line 97-99:

The most commonly used opioids in Sweden from 2012 to 2015 were oxycodone, tramadol and paracetamol combined with codeine.

Ref 9: Förskrivning av opioider i Sverige

Läkemdel doser och diagnoser (Prescription of opioids in Sweden, Drugs, doses and diagnoses.) Swedish medical products academy; 2020-02-17 [cited 2020-02-17]. Available from: <https://www.lakemedelsverket.se/globalassets/dokument/publikationer/lakemedelsprodukter-och-narkotika/forskrivning-av-opioider-i-sverige-2020-1.pdf>

And in Methods line 140-142:

ATC code N02A (opioids) includes, for example, morphine, oxycodone and tramadol, as well as combination drugs containing paracetamol, along with codeine.

The reference list has been corrected with one updated website.

Reference list:

11.	Socialstyrelsen. Läkemedelsregistret 2005. Available from: <https://www.socialstyrelsen.se/statistik-och-data/register/lakemedelsregistret/>.
