# Supplementary material for: Impact of hysterectomy on opioid use in patients with adenomyosis: A nationwide register study
Source: PLoS One. 2025 Jan 15;20(1):e0317135. doi: 10.1371/journal.pone.0317135 (PMC11734910; doi:10.1371/journal.pone.0317135)
Supplement: Attachment — Submitted filename: Comments by line Review Schmidt.docx [file pone.0317135.s008.docx]

Comments by line:

282 Patients with preoperative use of opioids/antidepressants and uterine size <300g were at increased risk for long-term opioid use.

292 ditto

293 opioid and antidepressant use

294 to 300 not correct! There is data that explains pain more severe in pts with smaller uteruses affected by1. Increased Uterine Contractility: Adenomyosis is associated with abnormal uterine contractility, including increased amplitude and decreased coordination of contractions. This dysperistalsis can lead to more intense pain, particularly in smaller uteri where the abnormal contractions may be more pronounced.[1]

2. Hyperinnervation: Adenomyotic lesions often exhibit increased nerve fiber density, particularly in the myometrium and at the margins of adenomyotic areas. This hyperinnervation can amplify pain perception, making symptoms more severe in smaller uteri where the concentration of nerve fibers may be higher relative to the uterine volume.[2][3]

3. Increased Lesional Production of Pain Mediators: Adenomyotic tissue produces higher levels of pain mediators such as prostaglandins and cytokines. In a smaller uterus, the relative concentration of these mediators may be higher, leading to more severe pain.[4]

4. Altered Uterine Peristalsis: Smaller uteri with adenomyosis may exhibit more significant alterations in uterine peristalsis, contributing to increased pain during menstrual cycles.[5]

These factors collectively contribute to the heightened pain experienced by patients with smaller uteri affected by adenomyosis.

References

[1] Rees CO, Thomas S, de Boer A, et al. Quantitative Ultrasound Measurement of Uterine Contractility in Adenomyotic vs. Normal Uteri: A multicenter Prospective Study. Fertility and Sterility. 2024;121(5):864-872. doi:10.1016/j.fertnstert.2024.01.009.

[2] Quinn MJ, Kirk N. Differences in Uterine Innervation at Hysterectomy. American Journal of Obstetrics and Gynecology. 2002;187(6):1515-9; discussion 1519-20. doi:10.1067/mob.2002.130007.

[3] da Cunha Vieira M, Andres MP, Riccio LGC, et al. Association of Uterine Tissue Innervation and Peripheral Nerve Density With Adenomyosis Related Pain. A Systematic Review. Reproductive Sciences (Thousand Oaks, Calif.). 2024;31(8):2137-2149. doi:10.1007/s43032-024-01587-8.

[4] Guo SW. Cracking the Enigma of Adenomyosis: An Update on Its Pathogenesis and Pathophysiology. Reproduction (Cambridge, England). 2022;164(5):R101-R121. doi:10.1530/REP-22-0224.

[5] Arena A, Zanello M, Orsini B, et al. Uterine Peristalsis in Women Affected by Adenomyosis: A Step Towards Functional Assessment. International Journal of Gynaecology and Obstetrics: The Official Organ of the International Federation of Gynaecology and Obstetrics. 2024;165(2):666-671. doi:10.1002/ijgo.15299. adenomyosis:

306 and subsequent use of opioids and antidepressants

314 malign poor choice change to “malignant” since using “benign” previously

321 63,8-69,6% change to 63.8-69.6% (comma inappropriate)

322 för change to for

322-325 “Considering the well-known ongoing opioid pandemic, an opioid usage of approx. 20% in this otherwise healthy group (>96% ASA 1-2) deserves attention.”

326…limitations. Should be additional limitations. (since they already mentioned some in the previous section)

332-335…”Therefore, we cannot conclude that the opioid use is due to pain caused by adenomyosis, but simply acknowledge that the use does not decline after hysterectomy.” This should include the antidepressants as well. Otherwise well stated.

337-338 “…not reduce need for opioid treatment among adenomyosis patients.” True for antidepressants as well.

351 … opioids/antidepressants and uterine size <300g.
